# Treatment Challenges in Multiple Sclerosis – A Continued Role for Glatiramer Acetate?

**DOI:** 10.3389/fneur.2022.844873

**Published:** 2022-04-15

**Authors:** Massimiliano Mirabella, Pietro Annovazzi, Wallace Brownlee, Jeffrey A. Cohen, Christoph Kleinschnitz, Christian Wolf

**Affiliations:** ^1^Fondazione Policlinico Universitario “A. Gemelli” IRCCS, Rome, Italy; ^2^Centro di Ricerca Sclerosi Multipla (CERSM), Università Cattolica, Rome, Italy; ^3^MS Center, ASST Valle Olona, Gallarate Hospital, Gallarate, Italy; ^4^Queen Square MS Centre, National Hospital for Neurology and Neurosurgery, London, United Kingdom; ^5^Department of Neurology, Mellen Center, Neurologic Institute, Cleveland Clinic, Cleveland, OH, United States; ^6^Department of Neurology, University Hospital Essen, Essen, Germany; ^7^Lycalis sprl, Brussels, Belgium

**Keywords:** disease modifying treatment, glatiramer acetate, special populations, multiple sclerosis, comorbidities

## Abstract

Earlier diagnosis, access to disease-modifying therapies (DMTs), and improved supportive care have favorably altered the disease course of multiple sclerosis (MS), leading to an improvement in long-term outcomes for people with MS (PwMS). This success has changed the medical characteristics of the population seen in MS clinics. Comorbidities and the accompanying polypharmacy, immune senescence, and the growing number of approved DMTs make selecting the optimal agent for an individual patient more challenging. Glatiramer acetate (GA), a moderately effective DMT, interacts only minimally with comorbidities, other medications, or immune senescence. We describe here several populations in which GA may represent a useful treatment option to overcome challenges due to advanced age or comorbidities (e.g., hepatic or renal disease, cancer). Further, we weigh GA's potential merits in other settings where PwMS and their neurologists must base treatment decisions on factors other than selecting the most effective DMT, e.g., family planning, conception and pregnancy, or the need for vaccination.

## Introduction

Glatiramer acetate (GA) was approved for the treatment of relapsing multiple sclerosis (MS) in 1996 in the US (1, 2), and 2001 in Europe (3), based on its beneficial effect on relapse rates in controlled trials (4–7). It is still widely prescribed as a safe and effective treatment after several million patient-years of exposure (8). Several generic alternatives have been developed (9, 10). GA is considered a platform therapy with modest effects on relapse-related clinical outcomes and no firmly established effect on delaying clinical progression or long-term disability (11). The continuing widespread use of this injectable agent despite newer, more efficacious DMTs may be attributable to its favorable safety profile with a lack of late adverse events and immunologic complications, or to its low level of interaction with comorbidities; however, part of the reason may also be the relatively few requirements for pre-treatment testing and on-treatment monitoring, the flexibility that it offers for family planning, or economic considerations. In this paper, we will discuss selected aspects and possible reasons for the enduring use of glatiramer acetate and its use in special populations of people with multiple sclerosis (PwMS).

MS, the most common chronic neuroinflammatory disease, causes demyelination, axonal degeneration, and gliosis, with focal inflammatory lesion activity usually predominating in the relapsing phase and diffuse inflammation and neurodegeneration becoming the main components for patient in the progressive phase (12). Although clinical distinctions are made between predominantly relapsing and progressive forms of MS (13, 14), the mechanisms underlying relapses and progression are present to varying degrees throughout the course of MS (13, 15–17). The prevalence of MS has increased in recent decades, and this may be due to the increasing sensitivity of radiographic methods and diagnostic criteria (18–22), and to longer survival (23).

Earlier diagnosis, access to disease-modifying therapies (DMTs), improved supportive care, and the fast-growing agency of PwMS have led to improvements in long-term outcomes (24–26). DMTs for MS suppress central nervous system (CNS) inflammation, reducing relapse rates and long-term disability. They work mainly during the predominantly relapsing phase by modulating the immune response, depleting immune cells or blocking their trafficking into the CNS (27). Important questions regarding treatment strategies include the optimal treatment approach in early MS, when and how to sequence treatments, including in patients with breakthrough activity, and when to de-escalate or discontinue treatment. The growing list of approved treatments for MS has made selecting the optimal agent for an individual patient more challenging. Current guidelines from the American Academy of Neurology (28), and European Committee for Treatment and Research in MS and the European Academy of Neurology (29) provide only limited guidance on starting, switching, and discontinuing, whereas treatment algorithms are provided in the National Health Service England guidelines (30), and in a recent position paper from the Multiple Sclerosis Treatment Consensus Group (17).

The two general approaches to treating MS involve either the early use of highly effective DMTs or the initial use of modestly effective DMTs, with escalation to highly effective DMTs when treatment response is inadequate (27, 31). Early highly-effective therapy maximizes anti-inflammatory effects early in the disease course, when they are most likely to be beneficial (32–35). Recent evidence from cohort studies suggests that early treatment with highly-effective therapy may be associated with a lower risk of disability progression (35, 36), and conversion to secondary progressive MS (34); however, this approach may expose some patients to an unnecessary risk of severe adverse effects such as infections, cardiac dysfunction, liver damage, or an increased risk of autoimmune diseases (37–40) (Table 1).

**Table 1:** US Food and Drug Administration contraindications, warnings and precautions for representative DMTs for MS^a^.

**DMT**	**Contraindications**	**Warnings and precautions**
**Modest efficacy**		
Interferons interferon beta-1a (41) interferon beta-1b (42)	History of hypersensitivity to natural or recombinant interferon beta, albumin or any other component of the formulation	Both agents: anaphylaxis and other allergic reactions; congestive heart failure, hepatic injury, seizures, thrombotic microangiopathy, monitoring for laboratory abnormalities interferon beta-1a only: depression, suicide, and psychotic disorders; decreased peripheral blood counts, autoimmune disorders interferon beta-1b only: depression and suicide, leukopenia, drug-induced lupus erythematosus, flu-like symptom complex, injection site necrosis and reactions
Glatiramer acetate (43)	Known hypersensitivity to glatiramer acetate or mannitol	Post-injection reaction, chest pain, lipoatrophy and skin necrosis, potential effects on immune response
Teriflunomide (44)	Severe hepatic impairment	Hepatotoxicity, embryofetal toxicity, bone marrow effects/immunosuppression potential/infections, hypersensitivity and serious skin reactions, peripheral neuropathy, increased blood pressure, respiratory effects, concomitant use with immunosuppressive or immunomodulating therapies
Monomethyl fumarate (45) Dimethyl fumarate (46) Diroximel fumarate (47)	Known hypersensitivity to monomethyl-, dimethyl-, or diroximel fumarate or any of the excipients; co-administration of any of these agents	All agents: lymphopenia, flushing anaphylaxis and angioedema, PML, liver injury Monomethyl fumarate only: herpes zoster and other serious opportunistic infections
**High efficacy**		
Natalizumab (48)	History of PML or a hypersensitivity reaction to natalizumab	PML, herpes infections, hypersensitivity/antibody formation, hepatotoxicity, immunosuppression/infections, laboratory test abnormalities, immunizations
Sphingosine-1-phosphate receptor modulators Fingolimod (49) Ponesimod (50) Ozanimod (51) Siponimod (52)	All agents: In the last 6 months, experienced myocardial infarction, unstable angina, stroke, transient ischemic attack, decompensated heart failure requiring hospitalization, or Class III or IV heart failure; Presence of Mobitz type II second-degree or third degree atrioventricular block, sick sinus syndrome, (or sino-atrial block for ozanimod), unless the patient has a functioning pacemaker; Fingolimod only: baseline QTc interval ≥ 500 msec, cardiac arrhythmias requiring anti-arrhythmic treatment with Class Ia or Class III anti-arrhythmic drugs, hypersensitivity to fingolimod or its excipients; Siponimod only: CYP2C9*3/*3 genotype; Ozanimod only: severe untreated sleep apnea; concomitant use of a monoamine oxidase inhibitor.	All agents: bradyarrhythmia and atrioventricular blocks/conduction delays, infections, macular edema, posterior reversible encephalopathy syndrome, respiratory effects, liver injury, fetal risk, severe increase in disability after discontinuation, increased blood pressure Fingolimod, ozanimod, siponimod: immune system effects after discontinuation Ozanimod, ponesimod, siponimod: unintended additive immunosuppressive effects from prior treatment with immunosuppressive or immune-modulating therapies Fingolimod and ponesimod: malignancies Fingolimod only: hypersensitivity reactions, PML
Alemtuzumab (53)	Infection with Human Immunodeficiency Virus.	Autoimmunity, infusion reactions, malignancies, immune thrombocytopenia, glomerular nephropathies, thyroid disorders, other autoimmune cytopenias, infections, acute acalculous cholecystitis, pneumonitis
Anti-CD20 B-cell depleting agents Ocrelizumab (54) Ofatumumab (55)	Both agents: active hepatitis B virus infection; Ocrelizumab only: history of life-threatening infusion reaction to ocrelizumab	Both agents: infusion reactions, infections, reduction in immunoglobulins, malignancies Ofatumumab only: fetal risk
Cladribine (56)	Current malignancy, pregnancy, breastfeeding, Human Immunodeficiency Virus infection, active chronic infections, hypersensitivity to cladribine.	Malignancies, risk of teratogenicity, lymphopenia, infections, hematologic toxicity, graft-vs.-host-disease with blood transfusion, liver injury, hypersensitivity, cardiac failure
*Mitoxantrone* (57)	Contraindicated in patients who have demonstrated prior hypersensitivity to it.	Cardiotoxicity, myelosuppression, secondary leukemia, potential human teratogen

a *Refer to updated local prescribing information for each agent for complete information*.

*PML, progressive multifocal leukoencephalopathy*.

In the traditional escalation approach, determining the optimal timing of escalation is challenging and requires balancing the need to allow adequate time for a therapeutic effect to manifest with the need for timely response to ongoing disease activity. A low threshold for escalation in the face of breakthrough activity may reduce future disability (58, 59). The criteria of “No Evidence of Disease Activity” (NEDA) has been proposed to guide tight control of MS activity (60, 61). This outcome focuses on inflammatory demyelination that causes *transient* disability, but it has limitations (62), and its use may not influence long-term progression (63), due to the underlying diffuse inflammation and neurodegeneration that appear to drive *long-term* disability (16, 63). While fully validated biomarkers to guide treatment decisions in MS are lacking, cerebrospinal fluid and plasma levels of neurofilament light chain reflect axonal damage in a wide variety of neurological disorders (64). Recently, this marker has shown promise for monitoring disease activity and response to therapy in PwMS (65).

Ongoing controlled studies comparing escalation and early highly-effective treatment strategies may help to identify the most effective approach (NCT03500328, NCT03535298).

Shared decision making is a theme that should guide the relationship between PwMS and their neurologists (66, 67). The growing number of available DMTs with different potential benefits and risks makes it difficult to identify the most appropriate treatment for each patient. Communication between patient and clinician can be suboptimal (68, 69). In the context of shared decision making, clinicians should contribute the medical basics for suitable treatments, considering drug properties, disease characteristics and other factors, e.g., comorbidities, while patients may express their informed preferences based on expected benefits and their personal risk tolerance (70).

## Glatiramer Acetate

**Figure 1:**
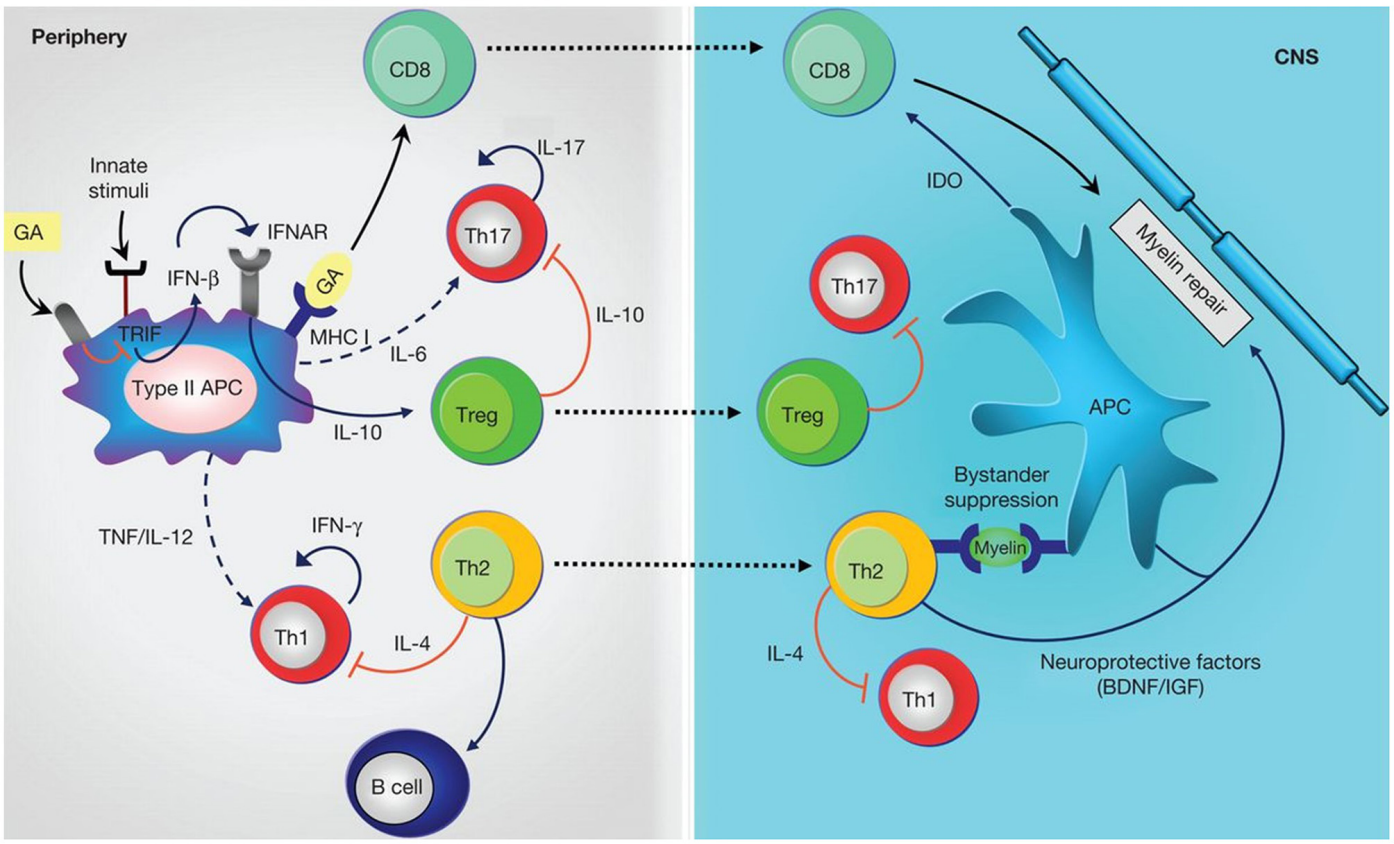
Anti-inflammatory mechanisms induced by glatiramer acetate (GA). GA treatment on antigen-presenting cells (APCs) leads to anti-inflammatory differentiation. Treatment modulates innate stimuli and is associated with down-regulation of type I interferon (IFN), increased T helper (Th)2, and regulatory T (Treg) cell differentiation. Reactivation of GA-reactive Th2 cells in periphery through presentation of myelin antigens is associated with bystander suppression. Th2 cells also modulate B-cell activation. Treg cells down-regulate secretion of proinflammatory cytokines by effector T (Teff) cells both in periphery and in the CNS. CD8+ T cells are generated by antigen presentation of GA in periphery and migrate to the CNS where they contribute to inhibiting myelin degradation. IL, Interleukin; TNF, tumor necrosis factor; IFNAR, interferon-receptor; MHC, major histocompatibility complex; BDNF, brain-derived neurotrophic factor; IGF, insulin-like growth factor; IDO, indoleamine-2,3-dioxygenase; solid lines, cytokines produced by the representative cells; dashed lines, reduced production of cytokines; red lines, inhibitory cytokines (72). [Figure from Prod92homme and Zamvil (72)].

Glatiramer acetate is an immunomodulating drug consisting of a complex polypeptide mixture (non-biological complex drug) obtained through the polymerization of the amino acids L-glutamic acid, L-alanine, L-lysine and L-tyrosine, followed by partial hydrolysis (43). It is administered by subcutaneous injection. Its mechanism of action is complex and not fully characterized but appears to involve effects on both innate and adaptive immune mechanisms. Briefly, it is thought to down-regulate myelin-specific T-cell activation and may compete with myelin basic protein peptides for binding to MHC class II molecules on antigen-presenting cells, leading to increased differentiation of T helper cells (Th)2, and T regulatory cells (Treg). Glatiramer acetate-reactive Th2 cells also suppress the activation of Th1 cells through “bystander suppression” and release neuroprotective factors, while the Treg cells reduce the secretion of proinflammatory cytokines by effector T cells. CD8+ T cells generated by antigen presentation of glatiramer acetate contribute to inhibiting myelin degradation (Figure 1) (71, 72).

## Special Populations That May Benefit From GA Treatment

### Patients With High Burden of Comorbidities

PwMS tend to have more comorbidities than the general population, and these can present specific challenges in treatment selection (73–75). The most common medical comorbidities include hyperlipidemia, hypertension, diabetes, gastrointestinal or thyroid diseases, which tend to increase with age (76). Diabetes, hypertension, and chronic obstructive pulmonary disease are associated with increased disability progression in PwMS (77). Vascular comorbidity correlates with poorer cognitive functioning and brain volume in PwMS (78).

Comorbidities increase the complexity of patient management by increasing the risk of drug-drug and drug-disease interactions. In addition to treatment for comorbidities, and for MS itself, PwMS often require pharmacotherapy to treat MS symptoms, such as fatigue, spasticity, pain, sleep disorders depression, urogenital, sexual and bowel dysfunction (79). A systematic literature review identified polypharmacy, defined as ≥ 5 prescription medications, in 15 to 59% of PwMS; rates increased with age, comorbidities, disability, cognitive deficits, and MS disease activity, and were associated with lower quality of life (80). Evidence to support treatment decisions in MS patients who have significant comorbidity is lacking. This knowledge gap can be attributed to the underrepresentation of such patients in clinical trials (81), limiting the possibility for evidence-based treatment. Thus, informed treatment decisions taking a patient's comorbidities and their accompanying essential medications into account rely mainly on empirical knowledge.

### Patients at Risk for Infections and Virus Reactivation

Infections represent a major cause of morbidity and mortality in the setting of MS (82, 83). PwMS are intrinsically prone to various infections such as urinary tract infections secondary to neurogenic bladder (84). Immunosuppressive DMTs may increase this risk (85, 86). All DMTs, except glatiramer acetate and interferons, impair immune surveillance to some degree (39, 40); however, it is important to distinguish between immunosuppressive drugs that impair immune function generally, increasing the risk for a broad range of infections, and immune modulating agents that selectively inhibit specific aspects of the immune system, thereby predisposing patient to a more restricted set of pathogens (87). There are continuing concerns about increased susceptibility to severe SARS-CoV-2 infection; however, PwMS have an infection risk similar to that of the general population (88). Among DMTs, the risk of infections in general is lower in patients receiving interferons and glatiramer acetate (89). Progressive multifocal leukoencephalopathy (PML), which is caused by emerging of neuropathogenic and neurovirulent pool of *John Cunningham virus* undergoing sequential genomic rearrangements in immunocompromised subjects, is also a major concern (90). Among DMTs for MS, natalizumab is associated with the highest risk for PML, but it also been reported very rarely with ocrelizumab, fingolimod, and dimethyl fumarate.

Evaluating the risk of infection has become one of the most important factors when choosing therapy or switching treatments (91), and monitoring for infections is an essential practice with some DMTs (Table 2).

**Table 2:** Infection screening required for disease-modifying treatments in MS.

**Disease-modifying drug**	**Basal infection screening**	**Infection monitoring**
Alemtuzumab	TBC, VZV, HPV, HBV, HCV, HIV	Pneumocystis, HSV, CMV, Listeria, Candida, HPV, HIV
Cladribine	TBC, VZV, HBV, HCV, HIV	HSV
Dimethyl fumarate	**-**	**-**
Fingolimod	TBC, VZV, HPV, HBV, HCV, HIV	Pneumonia, HSV, HPV, Cryptococcus
Glatiramer acetate	**-**	**-**
Interferons	**-**	**-**
Natalizumab	VZV, JCV, HIV	HSV, JCV
Ocrelizumab	TBC, VZV, HBV, HCV, HIV	HBV, HIV, JCV
Teriflunomide	TBC, HIV	**-**

*TBC, tuberculosis; VZV, varicella-zoster virus; HPV, human papillomavirus; HBV, hepatitis B virus; HCV, hepatitis C virus; HIV, human immunodeficiency virus; HSV, herpes simplex virus; CMV, cytomegalovirus; JCV, John Cunningham virus (human polyomavirus 2). (Source www.ema.europa.eu)*.

The risk of *hepatitis B virus* reactivation with glatiramer acetate is equal to that of the general population (92), and screening for latent tuberculosis is not required before prescribing glatiramer.

### Patients With Concomitant Liver Disease or History of Drug Induced Liver Damage

The risk of liver injury can limit a patient's treatment options. Several DMTs are associated with a risk of liver injury (alemtuzumab, fingolimod, interferons, mitoxantrone, teriflunomide) (93). Autoimmune hepatitis and reactivation of chronic liver infections can also occur during DMT treatment (93, 94). A retrospective Canadian study identified drug-induced liver injury in ~2% of MS patients treated with interferon beta (95). Baseline assessment of liver function is required for most DMTs, and several require periodic monitoring during treatment (Table 3).

**Table 3:** Liver function testing requirements for disease-modifying treatments in MS (94).

**Agent**	**Liver function screening tests**	**ALT monitoring**
**Injective treatments**		
Beta interferon	Yes	After 1, 3, 6 months and periodically thereafter
Glatiramer acetate	No (but suggested)	No
**Oral treatments**		
Fingolimod	Yes	After 1, 3, 6, 9, 12 months and bimonthly thereafter
Teriflunomide	Yes	Every 2 weeks for 6 months, then bimonthly
Dimethyl fumarate	Yes	Yes (suggested every 6 months)
Cladribine	Yes	No
**Infusional treatments**		
Natalizumab	Yes	Monthly for first 3 months, quarterly thereafter
Alemtuzumab	Yes	Monthly up to 48 months from last infusion
Ocrelizumab	Yes	No (but suggested every 6 months)

*ALT, alanine aminotransferase. [From Biolato et al. (94)]*.

Glatiramer acetate has a favorable overall liver safety profile (94). Sporadic reports of rare adverse liver effects with glatiramer acetate have included cases of suspected drug-induced liver injury and autoimmune hepatitis (93, 96), but no cases of *hepatitis B virus* or *hepatitis C virus* reactivation, or acute liver failure have been reported in patients treated with GA (94). Liver function testing is not required before initiating treatment with GA but the summary of product information suggests that patients be regularly monitored for signs of hepatic injury and instructed to seek immediate medical attention in case of symptoms of liver injury (97).

### Elderly Patients

Peak MS incidence occurs in early adulthood, but prevalence peaks in late adulthood. Currently, the peak prevalence of PwMS is estimated to be between age 50 and 60 years (98, 99), meaning that many PwMS are older than the patient populations in pivotal trials for DMTs. The prevalence of MS in the elderly is increasing due to population aging, earlier diagnosis, access to DMTs, and improved supportive care (18–23). Moreover, about 5% of patients present with late-onset MS (onset at ≥ 50 years), often with motor dysfunction and a relatively poor prognosis (100–102).

The increased rate of comorbidities, with the accompanying polypharmacy, and immune senescence in elderly PwMS make selection of the optimal agent even more challenging. There is evidence that inflammatory lesion activity decreases with age in PwMS (103), and that the efficacy of DMTs decreases as well (28). There is no evidence to support differences in efficacy among DMTs in elderly patients (104). Meanwhile, some side effects of highly effective DMTs are more common/serious in elderly patients (105) (Table 4); therefore, the benefit-risk of a DMT may change as patients age, favoring a less effective DMT with a lower risk for adverse effects.

**Table 4:** High impact adverse events of DMTs and correlation with increasing age (105).

**Drug**	**Most important adverse events**	**(Potentially) increased** **risk with age**	**References**
Natalizumab	Occurrence of PML Fatal cases of PML HSV1/VZV reactivation Neutralizing antibodies	(↑) ↑ (↑)	(106) (107) (108)
Fingolimod	Bradycardia Arterial hypertension HSV1/VZV reactivation PML Skin malignancies (BCC) HPV infection	↑ ↑ ↑ (↑) ↑	(109) (110) (111)
Alemtuzumab	HSV1/VZV reactivation Listeriosis, candidiasis, nocardiosis Secondary autoimmune diseases	↑ ↑	(112) (113) (114)
Cladribine	HSV1/VZV reactivation Tuberculosis Solid malignancies	↑ ↑	(115)
Ocrelizumab	HSV1/VZV reactivation HBV Hypogammaglobulinemia Breast cancer PML (carry over)	↑ (↑)^a^ ↑ (↑)	(116)

*↑ Generally higher risk with higher age independent of DMT; (↑) potential higher risk with higher age and use of DMT*.

a *Rituximab-associated cases of serious infections reported with higher age*.

*BCC, basal cell carcinoma; DMT, disease-modifying therapy; HBV, hepatitis B virus; HPV, human papilloma virus; HSV1, herpes simplex virus; PML, progressive multifocal leukoencephalopathy; VZV, varicella zoster virus*.

*[modified from Schweitzer et al. (105) with addition of secondary autoimmunity with alemtuzumab-reference EMA, Lemtrada EPAR added]*.

The pathological mechanisms underlying neurodegeneration during the progressive phase are thought to involve compartmentalized inflammation driving neurodegenerative tissue responses to chronic inflammatory injury (117). These mechanisms driving progressive disease do not appear to respond to DMT. Unless there is evidence of active inflammation, a high risk/high efficacy DMT might not be the right choice.

The absence of effective treatment or neuroprotective strategies for progressive disease in elderly patients, combined with the observed reduction in the efficacy of DMTs in the elderly (32, 104, 118), suggest a need to consider carefully the benefit-risk profile in this population (6). Thus, one of the unique challenges when treating elderly PwMS is determining the appropriateness/timing of DMT de-escalation or discontinuation in stable patients without clinical or radiological disease activity (119, 120). In a recent survey of 377 MS patients age ≥ 45 years who had been receiving DMT for ≥ 5 years, only 12% reported that they would consider discontinuing DMTs if they had no evidence of disease activity (121). Predictors of relapse/rebound included younger age, female sex, moderate disability, and a relapse within 1 year of discontinuation (122). Reactivation of MS disease activity after discontinuation of DMTs is independently associated with age at discontinuation, MRI activity at discontinuation, and the duration of clinical stability (123).

### Pregnancy and Family Planning

The typical age at MS onset overlaps with childbearing years. Pregnancy is associated with lower MS disease activity (124), and may provide natural protection when DMT is suspended. Ideally, all agents except interferon or GA should be discontinued before attempting conception. An increasing number of pregnancies are conceived in women who are receiving DMT. Depending on the DMT, discontinuation in this situation may result in increased disease activity (125, 126). However, most of the safety data on exposure come from the first month after conception and focus on teratogenic risks and do not cover late term complications e.g., due to immunologic effects (126, 127). MS patient registries show that the injectable DMTs glatiramer acetate and interferon beta are indeed safe before conception and in patients with first trimester exposure, although limited data are available on their continuation throughout pregnancy (128–130). In light of the favorable safety evidence, both the US Food and Drug Administration and the European Medicines Agency have removed the restriction on GA use in pregnancy; however, as a precaution it is generally preferred to avoid exposure during pregnancy unless the benefit to the mother outweighs the risk to the fetus. When deemed necessary, administering a bridging therapy with a safer agent can provide coverage while trying to conceive (126). Switching treatments when planning a pregnancy in clinically stable patients is common practice (131); however, bridging therapy must be initiated early enough to be effective during the first trimester, when relapse risk is highest. Other strategies may include administering highly effective therapies that have long effect durations (e.g., ocrelizumab, alemtuzumab, cladribine) before pregnancy while observing the appropriate washout periods (126) or leveraging the fact that monoclonal antibodies do not cross the placenta until the second trimester (132).

Recent data from the German MS and Pregnancy Registry showed no evidence of adverse effects of GA exposure during breastfeeding on infant development, hospitalization, or the use of antibiotics (133). This has led to removal of the restriction on GA use while breastfeeding (134).

### Vaccination

Vaccination does not appear to increase MS disease activity (135, 136). On the contrary, a variety of vaccine-preventable infections can exacerbate disease activity and trigger relapses (137–140); therefore, vaccination against preventable infections, for example influenza, can improve disease control. Guidelines from the American Academy of Neurology recommend following local vaccination schedules unless vaccination is contraindicated (e.g., PwMS already receiving immunosuppressive or immunomodulating therapy) or the patient is experiencing a relapse. In relapsing patients, vaccination should be delayed until resolution or until the relapse is no longer active or progressing (141). Moreover, PwMS should undergo a vaccination status assessment and updating of vaccinations soon after MS diagnosis to carefully plan and administer vaccinations early in the course of MS, before starting DMT (141, 142). Seroconversion after vaccination is attenuated in patients receiving anti-CD20 therapy, and the response to novel antigens (not encountered previously in life) is weakened (143), including after COVID-19 mRNA vaccination (144). Vaccines are less effective in the elderly, and immunodepleting therapy may further reduce the response to vaccines in this population.

In PwMS receiving GA, seroconversion was lower after the 2009 H1N1 pandemic influenza vaccine (*n* = 37) and the 2010 seasonal influenza vaccine (*n* = 12), compared to healthy controls (145). Because of this observation, the 2019 American Academy of Neurology guidelines stated that GA is “possibly” associated with a reduction in vaccine response. However, in a study of seroconversion after the 2010/2011 and 2011/2012 influenza vaccines, patients receiving GA (*n* = 26) had normal post-vaccination seroconversion rates for the 3 influenza antigens (H1N1 88.5%, H3N2 73.1%, B strain 80.8%, *n* = 26) (146). Response to the 2012/2013 seasonal influenza vaccine in patients receiving GA (*n* = 23) was also similar to healthy controls after 3, 6, and 12 months (147). Moreover, seroconversion after vaccination against SARS-CoV-2 is not attenuated in patients receiving GA (148–150). Live vaccinations are contraindicated in people receiving DMTs, except GA. Most oral DMTs interfere with response to *hepatitis B virus* vaccinations, whereas injectables therapies do not (151).

## Conclusions

Given the difficulty of predicting the long-term course of MS at diagnosis, and although the early use of higher efficacy therapies may be warranted to prevent long-term disability, especially in patients with highly active disease, GA may be considered in scenarios where high efficacy therapies would pose more risk. GA may be appropriate later in the disease course in response to evolving patient conditions (e.g., aging, accumulating comorbidities, chronic treatment with corticosteroids and other immunosuppressants), or as a bridging therapy during conception, pregnancy, and breastfeeding. Similarly, it may be useful for vaccinations strategies, e.g., use of live or attenuated vaccines as well as vaccines against hepatitis B virus or SARS-CoV-2.

## Author Contributions

All authors listed have made a substantial, direct, and intellectual contribution to the work and approved it for publication.

## Funding

This study received funding from Viatris Inc. The funder was not involved in the study design, collection, analysis, interpretation of data, the writing of this article or the decision to submit it for publication.

## Conflict of Interest

MM received consulting and/or speaking fees, research support or travel grants from Almirall, Bayer Schering, Biogen, CSL Behring, Sanofi-Genzyme, Merck, Novartis, Teva, Roche, Viatris (Mylan). JC received personal compensation for consulting for Biogen, Bristol-Myers Squibb, Convelo, Genentech, Janssen, NervGen, Novartis, and PSI; speaking for H3 Communications; and serving as an Editor of Multiple Sclerosis Journal. CW is a partner at Lycalis sprl. His organization has received compensation for consulting and speaking from Viatris (Mylan), Merck KAaG, Roche, Immunic, BMS Celgene, Novartis, Teva, Synthon, 2BBB, ICON, and Desitin. WB has received speaker honoraria and/or participated in advisory boards for Biogen, Celgene, Merck, Novartis, Roche, Sanofi and Viatris. PA received honoraria for lecturing and/or participation in advisory boards, and/or travel expenses for attending congresses and meetings from Almirall, Biogen, BMS-Celgene, Merck, Novartis, Roche, Sanofi-Genzyme, Teva Italia, and Viatris. CK received speaker honoraria and/or participated in advisory boards for Alexion, Biogen, Celgene, CSL Behring, Janssen-Cilag, MedDay, Merck, Novartis, Roche, Sanofi Genzyme, Teva and Viatris.

## Publisher's Note

All claims expressed in this article are solely those of the authors and do not necessarily represent those of their affiliated organizations, or those of the publisher, the editors and the reviewers. Any product that may be evaluated in this article, or claim that may be made by its manufacturer, is not guaranteed or endorsed by the publisher.
